# Acute intracranial EBV and CMV infections after chemoimmunotherapy for EBV-associated nasopharyngeal carcinoma: a case report and literature review

**DOI:** 10.3389/fonc.2025.1608787

**Published:** 2025-10-14

**Authors:** Zhenyu Zhang, Jiong Lin, Xinming Song, Xiaoqiong Yi, Hechao Zhou, Zumin Xu

**Affiliations:** Cancer Center, Affiliated Hospital of Guangdong Medical University, Zhanjiang, Guangdong, China

**Keywords:** nasopharyngeal carcinoma, trigeminal neuralgia, acute intracranial EBV and CMV infections, case report, ganciclovir

## Abstract

A 36-year-old male patient presented to our hospital complaining of epistaxis for 3 months and persistent headaches with facial numbness for 3 days. After a series of exams, he was diagnosed with nasopharyngeal carcinoma (T4N2M1, stage IVB, AJCC 8th), with a biopsy consistent with non-keratinizing squamous cell carcinoma, and received a combination therapy of gemcitabine, cisplatin, and tislelizumab. Following the first dose, headaches and facial numbness were relieved. On the third day, however, he developed recurrent fever, with a peak body temperature of 39.2 °C, and developed severe paroxysmal stabbing pain in the right frontal region suggestive of trigeminal neuralgia, along with numbness on the right face. We considered multiple possibilities and provided symptomatic treatments, but with poor efficacy. Subsequently, given the emergence of prominent neurological symptoms and fever, we proceeded with a lumbar puncture for cerebrospinal fluid (CSF) analysis. Metagenomic next-generation sequencing (mNGS) of CSF detected the presence of Epstein-Barr virus (EBV) and cytomegalovirus (CMV), and acute intracranial viral infections were considered. After treatment with ganciclovir, the patient’s body temperature returned to normal, and headaches and facial numbness were alleviated, and no pathogens were detected in a follow-up examination. We report a case of trigeminal neuralgia emerging post-chemoimmunotherapy, accompanied by CSF positivity for EBV and CMV, where antiviral intervention with ganciclovir resulted in significant symptom alleviation.

## Introduction

Nasopharyngeal carcinoma is one of the common malignant tumors in China, most of which are caused by EBV infection. The diagnosis and staging of nasopharyngeal carcinoma are mainly conducted through imaging and pathological assessments. Its treatment plans primarily consist of chemotherapy, radiotherapy, and immunotherapy. The patient in this case presented with distant metastasis at initial diagnosis, fitting into a category of recurrent or metastatic nasopharyngeal carcinoma (RM-NPC), and was suitable for treatment with tislelizumab in combination with gemcitabine and cisplatin (1). The EBV belongs to the herpesviridae family, subfamily γ-herpesvirinae, and has a tropism for human B lymphocytes and nasopharyngeal epithelial cells. In rare cases, it infects T lymphocytes or natural killer (NK) cells, which may lead to persistent active infections. At present, many studies have shown that EBV is closely related to the occurrence and development of nasopharyngeal carcinoma. Additionally, CMV is a ubiquitous β-herpesvirus with a broad pathogenic spectrum in humans.

As far as we know, there have been few reports to date of EBV and CMV detection in the CSF of nasopharyngeal carcinoma patients. The development of trigeminal neuralgia following chemoimmunotherapy is also exceedingly rare. This case suggests that trigeminal neuralgia may be associated with acute EBV and CMV infections. Furthermore, the patient’s favorable outcome provides a reference for using ganciclovir as a treatment strategy in similar future cases.

## Case presentation

In March, 2024, a 36-year-old male presented to our hospital complaining of epistaxis for 3 months and persistent headaches with facial numbness for 3 days. The patient exhibited a Karnofsky Performance Status (KPS) score of 90 and Eastern Cooperative Oncology Group (ECOG) performance status of 0. His vitals were stable, with initial readings showing a temporal temperature of 36.2°C, respiratory rate of 20, heart rate of 72, and blood pressure of 99/69. His physical exam demonstrated bilateral enlarged superficial cervical lymph nodes, and demonstrated hypoesthesia in the trigeminal nerve (CN V) distribution: diminished light touch sensation was observed in the ophthalmic division (V1: forehead and upper eyelid), maxillary division (V2: midfacial region), and mandibular division (V3: chin and mandibular area). The patient had no significant past medical history, with no personal or family history of cancer and no psychiatric conditions.

**Figure 1:**
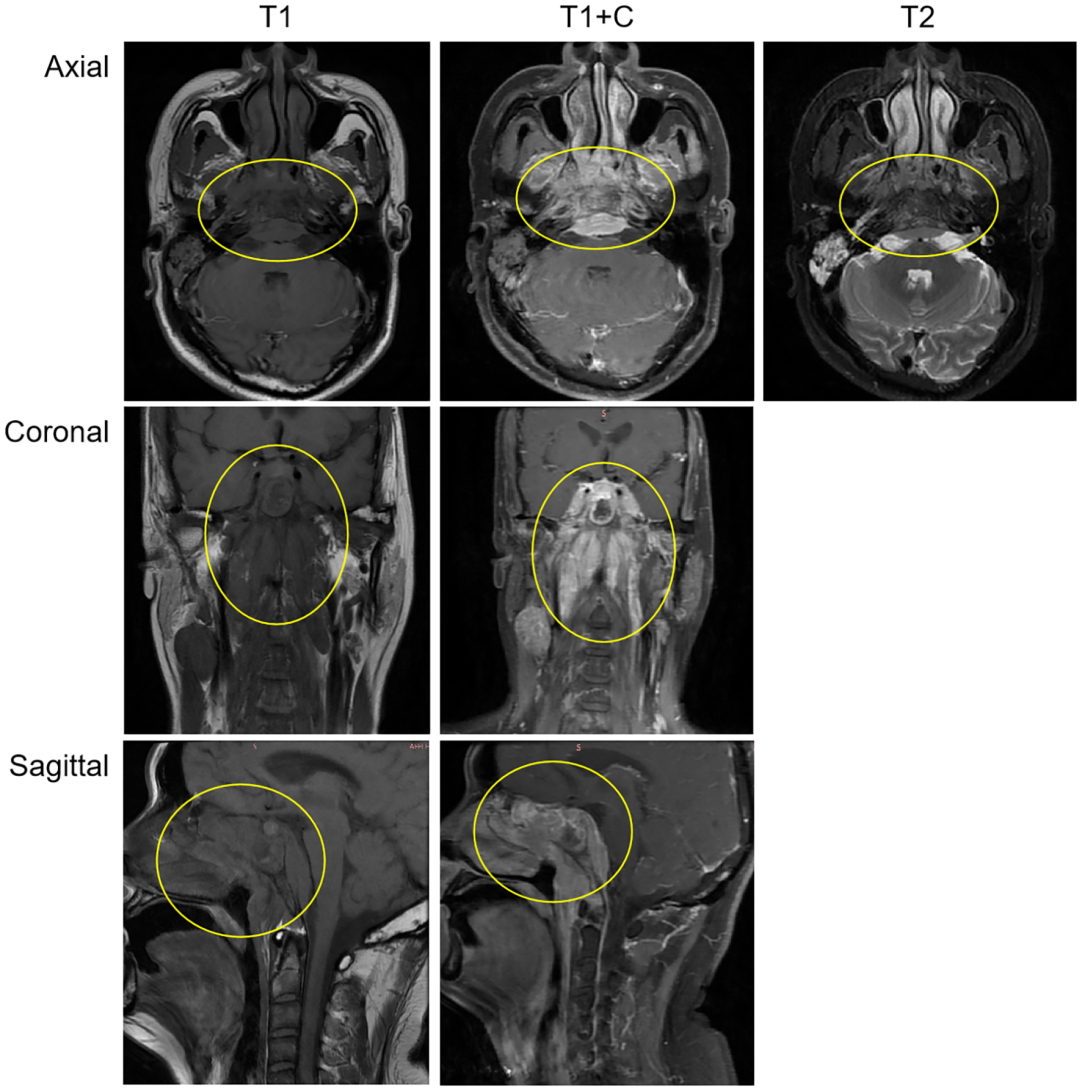
The MRI demonstrated a soft tissue mass measuring 38 mm×20 mm in the largest axial dimension, which showed isointensity signal on T1 WI and slightly hyperintense signal on T2W with significant enhancement after contrast-enhanced scan. The mass involves the surrounding normal tissue. The findings suggest nasopharyngeal carcinoma; Multiple enlarged lymph nodes, of varying sizes, were observed in the retropharyngeal space and bilateral cervical regions, suggestive of metastatic involvement.

After admission, relevant auxiliary examinations were carried out. Serum tests on March 19, 2024 revealed an elevated cytokeratin 19 fragment (35.600 ng/mL). An EBV-DNA test (whole blood) was performed on March 20 (< 500 copies/mL). Nasopharyngeal and cervical magnetic resonance imaging (MRI) findings from March 20 indicated nasopharyngeal carcinoma with multiple lymph node metastases (**Figure 1**). The MRI demonstrated mucosal thickening in the nasopharyngeal roof and posterior wall with involvement of the lateral pharyngeal recesses. A soft tissue mass measuring 38 mm×20 mm in the largest axial dimension was identified, which showed isointensity signal on T1 WI and slightly hyperintense signal on T2W with significant enhancement after contrast enhanced scan. Obliteration of the bilateral pharyngeal recesses and eustachian tube orifices was observed. The lesion demonstrated extensive local invasion with encroachment into the parapharyngeal space, pharyngobasilar fascia, etc. Multiple enlarged lymph nodes ranging from small to large were identified in the retropharyngeal space and bilateral cervical levels II; through III and Va, some of which merged into masses. The dominant lymph node, located in the right level III, measured 26 mm×27 mm×35 mm in maximal dimensions. Positron emission tomography-computed tomography (PET-CT) demonstrated a standardized uptake value maximum (SUVmax) of 8.76 in the nasopharyngeal soft tissue mass, with SUVmax values of 7.52 in the bilateral parapharyngeal and cervical lymph nodes and 4.86 in the pulmonary nodule (**Supplementary Figure 1**).

**Figure 2:**
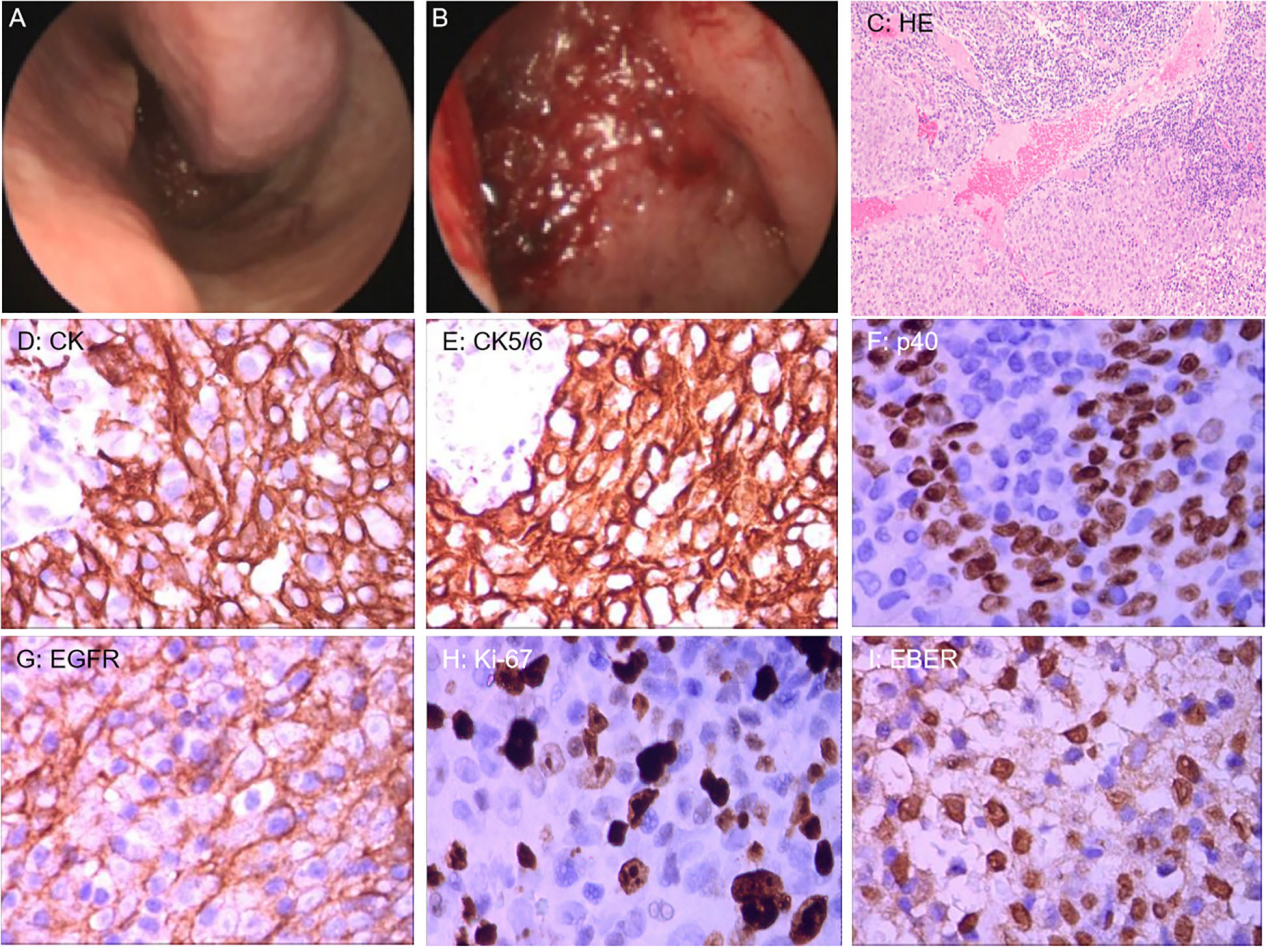
**(A, B)** Nasal endoscopic examination revealed mucosal thickening of the nasopharyngeal roof and posterior wall. **(C)** Hematoxylin and Eosin staining (100x) revealed tumor cells with nested growth in abundant lymphoid tissue. **(D–H)** Immunohistochemical staining (400x): CK and CK5/6 showed diffuse and strongly positive expression in the cytoplasm of tumor cells; p40 showed strongly positive expression in the nuclei of tumor cells; EGFR showed positive expression on the membrane of tumor cells; Ki-67 showed positive expression in the nuclei of tumor cells. **(I)** Epstein-Barr virus-encoded RNA (EBER) in situ hybridization (400x) revealed positive expression in the nuclei of tumor cells.

On March 21, endoscopic-guided nasopharyngeal mass biopsy was performed, with histopathological examination confirming non-keratinizing squamous cell carcinoma. Immunohistochemical staining demonstrated positivity for CK, CK5/6, p40, and EGFR, with Ki-67 at approximately 80%. Additionally, *in situ* hybridization revealed positivity for EBER (**Figure 2**).

**Figure 3:**
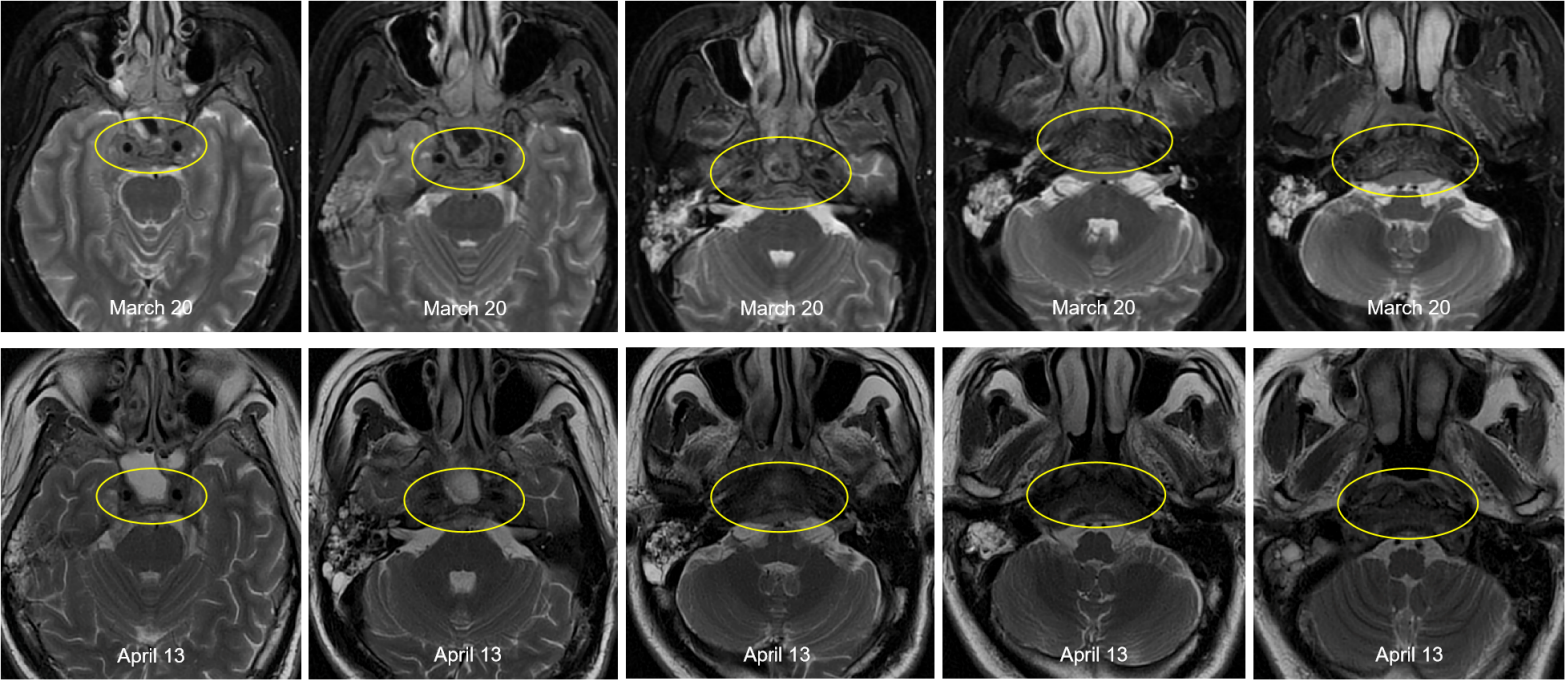
Comparison of pre- and post-treatment imaging showed a slight reduction in tumor size following chemoimmunotherapy.

This patient was diagnosed with nasopharyngeal non-keratinizing squamous cell carcinoma (T4N2M1, stage IVB, AJCC 8th), which had indications of palliative chemoimmunotherapy, and we obtained informed consent from patients and families for all of our treatments. Therefore, the planned therapeutic regimen consists of gemcitabine (at a dose of 1 g/m2 on days 1 and 8) and cisplatin (40 mg/m2, days 1-2), plus tislelizumab (200 mg on day 1). Alleviation of reported symptoms was observed following the first dose, potentially reflecting treatment-induced tumor volume reduction and subsequent decreased mass effect on adjacent neural structures. But, on day 3 post-treatment, i.e. on March 29, the patient developed recurrent fever, peaking at 39.2 °C. He also developed severe paroxysmal stabbing pain in the right frontal area suggestive of trigeminal neuralgia, accompanied by increased numbness on the right face. The NRS pain score was 9. His labs were notable for high C-reactive protein (CRP) at 117.46mg/L. His blood culture results (e.g., bacteria and fungi) were negative. The fever is considered to be caused by a bacterial infection (gemcitabine administration on day 8 of the first cycle was withheld due to fever); however, it persisted despite antibiotic therapy, which included cefoperazone and ceftriaxone. After administration of dexamethasone, symptoms were not relieved, and morphine failed to provide adequate analgesic effect. To assess for pulmonary infection and evaluate whether tumor progression is contributing to the clinical deterioration, chest CT and cranial MRI were performed. The CT (April 6) revealed no significant infectious foci (**Supplementary Figure 2**). The MRI (April 13) demonstrated a slight reduction in tumor size compared to previous imaging (**Figure 3**).

**Figure 4:**
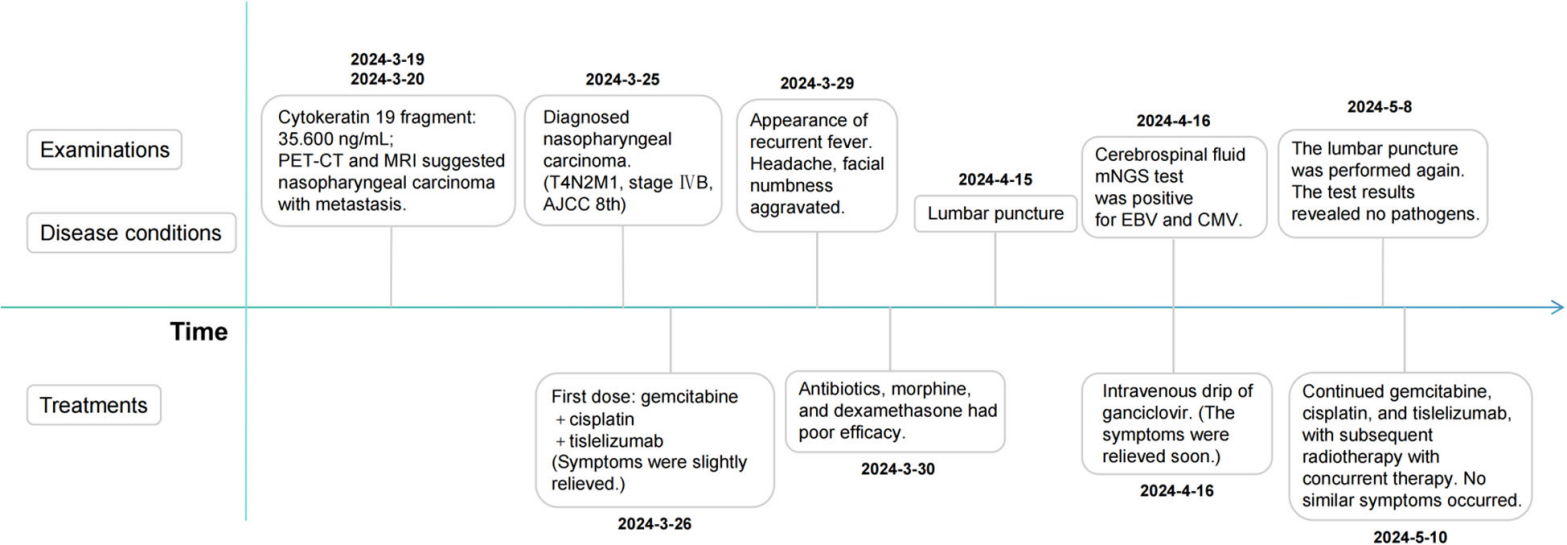
Timeline of the patient’s symptoms, examinations, diagnosis and treatments.

Respiratory viral infections were excluded through some laboratory examinations. We speculate that the symptoms of fever, headaches, and facial numbness are most likely caused by intracranial infection, but the causative pathogen remains unidentified. Therefore, lumbar puncture was performed on April 15th, and CSF was taken for test. Routine examinations of CSF include Pandey’s test (2+), total number of cells (590×10^6/L), white blood cells (510×10^6/L), chlorine (118.70mmol/L), glucose (2.00mmol/L), micro-albumin (1144.80mg/L), micro-total protein (2534.90mg/L). The mNGS test of CSF was positive for EBV (normalized sequence counts: 96) and CMV (normalized sequence counts: 174). Besides, a blood test dated April 11th revealed a reduction in absolute T-cell counts, suggesting an impairment in immune function. Then the antiviral therapy with ganciclovir (0.25 g, ivgtt, q12h) was initiated based on the recommendation of the infectious disease physician. The antiviral treatment exhibited a significant therapeutic effect. One day post administration, the body temperature returned to normal, and the pain and facial numbness symptoms were markedly alleviated. The examination on April 29th indicated a return to normal levels of T-cell counts. On May 8th, a follow-up examination of the CSF revealed that mNGS indicated no pathogens were detected. During the subsequent eight months of tumor treatment, the patient did not experience the aforementioned symptoms again (**Figure 4**).

The patient received chemoimmunotherapy (treatment plan as before) from May 10th to August 22nd, completing cycles 2 through 6, and achieved a complete response (CR) after the sixth cycle (**Supplementary Figure 3**). Radiotherapy of the nasopharynx and neck, delivered via VMAT using 6-MV X-rays with a prescribed dose of 70 Gy in 33 fractions to the PGTVnx, was administered from October 22 to December 23, along with concurrent chemotherapy, immunotherapy, and targeted therapy. As of December 2024, the therapeutic response was evaluated as CR (**Supplementary Figure 4**), and the patient is alive with no evidence of disease at last follow-up (August 2025). All treatments mentioned above were conducted with the informed consent of the patient and family members.

## Discussion and literature review

We encountered a case in which EBV may play a critical pathogenic role. EBV, a γ-herpesvirus with recognized oncogenic potential, is ubiquitous in the human population, with an infection prevalence approaching 90%, as documented in previous literature (2, 3). Within the majority of immunocompetent hosts, EBV establishes a subclinical, lifelong infection of B lymphocytes and nasopharyngeal epithelial cells (4). This virus exhibits cell-specific entry mechanisms. For B cell infection, EBV utilizes gp350/220 to engage complement receptor CD21, initiating clathrin-dependent internalization (5). Most T and NK cells do not express the EBV receptor CD21. However, CD21 receptor transfer from B cells to T or NK cells via immunological synapses may facilitate EBV infection in T or NK cells (6). Additionally, EBV may induce CD40 expression on infected cells, enabling interaction with CD40 ligand on T cells, thereby promoting EBV infection in T or NK cells (7). Following infection, activation of the co-stimulatory receptor CD137 on T or NK cells further enhances their proliferation (8). These mechanisms may explain EBV-associated lymphoproliferative disorders involving T or NK cells. Furthermore, EBV infects nasopharyngeal epithelial cells, and drives EBV-associated nasopharyngeal carcinoma by multifactorial collaborations. In epithelial cells, viral tropism is mediated by high-affinity binding of glycoprotein gH/gL to Ephrin A2 (EPHA2), facilitating receptor-mediated endocytosis (9). After infecting epithelial cells, the virus cause oncogene activation and oncosuppressor gene inactivation by inducing epigenetic changes in the host genome, as well as evading immune clearance by interfering with immune surveillance mechanisms (10). And EBV is mainly in the state of latent infection in nasopharyngeal carcinoma, and its expression of latent proteins promotes cell proliferation and survival by regulating host signaling pathways, among which latent membrane protein 1 (LMP1) can simulate CD40 receptor, and then continuously activate NF-κB and STAT3 signaling pathways, promote cell proliferation, inhibit apoptosis, and induce epithelial-mesenchymal transformation (EMT) (11). In this case report, EBV association was confirmed by EBER *in situ* hybridization.

A patient with carcinoma presented with cardinal symptoms of headaches and facial numbness, which were considered attributable to the tumor. These symptoms were alleviated after chemoimmunotherapy, suggesting a favorable therapeutic response. However, by the third day, his condition did not improve as anticipated. He began to develop recurrent fever, with a marked exacerbation of headaches and facial numbness compared to prior. Initially, we were more inclined to attribute the relapse of symptoms to tumor progression. But the MRI on April 13th revealed a reduction in tumor size after chemoimmunotherapy. Other than tumors, several causes can lead to such symptoms. Inflammatory reactions may occur after chemotherapy as chemotherapeutic agents can activate them, leading to neuralgia and a series of pathological changes, and this possibility should not be overlooked (12, 13). Chemotherapeutic agents damage the somatosensory nervous system by inducing neuroinflammation and oxidative stress, resulting in the sensitization of neurons. This is a direct mechanism of chemotherapy-induced neuropathic pain, and neuroinflammation is described as one of the initiating factors of neuropathic pain. Furthermore, immunotherapy can also cause neurological damage. PD-1 inhibitors, such as tislelizumab, may lead to overactivation of the immune system by blocking the PD-1 signaling pathway. In this context, self-tolerance of the nervous system may be disrupted, thereby potentially causing nerve damage (14, 15). The PD-1 pathway typically functions to inhibit immune responses and prevent excessive inflammation. When this pathway is blocked by inhibitors, the immune system may become dysregulated, potentially leading to autoantibody production, complement activation, and enhanced inflammation. But such neurological damage events are rare in clinical practice. Moreover, following inhibition of the PD-1 pathway, the expression of pro-inflammatory cytokines (such as TNF and IL-6) in nerve tissue may increase, which may promote inflammatory responses and directly damage nerve cells; this mechanism is particularly evident in chemotherapy-induced neuropathy, in which PD-1 inhibitors may exacerbate chemotherapy-related neuropathic pain, manifesting as mechanical allodynia and chronic neuropathy (14, 16). The onset time of PD-1 inhibitor-associated neuritis typically occurs within a short period after treatment initiation. Research indicates that symptoms usually appear within the first three months after initiation of Immune Checkpoint Inhibitors (ICIs) and progress rapidly (17). Symptoms usually improve after discontinuation of ICIs. In severe cases, early use of glucocorticoids can promote recovery. However, for the patient in this case, glucocorticoid treatment did not relieve the symptoms. It’s worth noting that neuroinflammation and neurological damage induced by tumor progression and chemoimmunotherapy can both lead to headaches and facial numbness, but their commonality is that antiviral therapy cannot alleviate the symptoms. Regarding the fever, respiratory infections were excluded through a series examinations.

To figure out the cause of the patient’s deteriorating symptoms, we performed mNGS of the CSF. The results revealed the presence of both EBV and CMV. Then, we considered that the aforementioned symptoms might be caused by intracranial viral infections and implemented antiviral treatment based on the examination results of the CSF. The marked alleviation of fever, headaches and facial numbness following ganciclovir therapy further confirmed our hypothesis.

Current literatures underscore the importance of excluding chronic active EBV infection (CAEBV) in patients with persistent EBV latency who develop unexplained symptoms of infections. CAEBV is characterized by clonal proliferation of EBV-infected T cells, NK cells, or B cells in individuals with prolonged EBV latency, presenting with prolonged or recurrent infectious mononucleosis (IM)-like symptoms and potential multi-organ involvement (18). The revised diagnostic criteria in 2022 suggested that the following four criteria should all be met before CAEBV can be diagnosed: Persistent or recurrent IM-like symptoms for more than 3 months; Detection of an increased number of EBV genomes in peripheral blood and/or affected tissues; Detection of EBV-infected T or NK cells in peripheral blood and/or affected tissues; Chronic illness that cannot be explained by other known disease processes at the time of diagnosis (19). Moreover, the revised diagnostic criteria emphasize that confirmation of elevated number of EBV genomes and EBV-infected T or NK cells are required for the diagnosis of CAEBV. The patient did not meet the diagnostic criteria for CAEBV, but notably, should the patient develop unexplained persistent fever or multi-organ EBV involvement in the future, re-evaluating for CAEBV through a comprehensive diagnostic workup would be warranted.

The trigeminal neuralgia in this case is likely due to acute intracranial viral infection caused by viral reactivation under immunosuppression and immunosuppressed state may be attributed to chemotherapy (20). Both gemcitabine and cisplatin are potent myelosuppressive agents that significantly reduce absolute counts of T cells, B cells, and neutrophils in peripheral blood, impairing immune surveillance. Gemcitabine also directly kills proliferating lymphocytes by inhibiting DNA synthesis (21), and cisplatin induces DNA crosslinking damage and apoptosis, further compromising immune cell function. Under such immunosuppression, EBV may reactivate into the lytic cycle and invade the central nervous system (CNS). PD-1 inhibitors (e.g., tislelizumab) enhance T-cell activation and proliferation, but it cannot be completely denied that it is not associated with immunosuppression.

In clinical practice, trigeminal neuralgia occurring after chemoimmunotherapy has been rarely reported. Instances of acute intracranial EBV infection in patients with EBV-associated nasopharyngeal carcinoma are also exceedingly rare and previous studies retrieved by us demonstrated that EBV-positive CNS diseases are uncommon, with reported incidences of only 5.2% and 9.7% in studies (22, 23), underscoring the rarity of such events (4, 24). In addition, CMV is a ubiquitous DNA virus that can cause infection and result in 40-100% seropositivity (25, 26). Although CMV infections are very common, most infections are asymptomatic or mild, presenting as benign and self-limiting processes. Reactivation or infection of CMV is also primarily considered in immunocompromised patients, such as solid organ or hematopoietic cell transplant recipients, HIV-infected patients, and patients undergoing cancer chemotherapy, which can lead to severe disease (27, 28).

## Conclusion

Here, we report a rare case in which a patient with EBV-associated nasopharyngeal carcinoma developed trigeminal neuralgia after treatment with gemcitabine, cisplatin, and tislelizumab. This symptom may be associated with acute intracranial EBV and CMV infections. After discontinuation of chemoimmunotherapy and administration of ganciclovir, the symptom was alleviated, and no viral positivity was detected in a follow-up examination of CSF. During subsequent treatments including chemoimmunotherapy, radiotherapy, and targeted therapy, no similar symptoms manifested. This case may provide a valuable treatment strategy for similar patients in the future.

## Data Availability

The original contributions presented in the study are included in the article/**Supplementary Material**. Further inquiries can be directed to the corresponding authors.
