# Supplementary material for: Acute intracranial EBV and CMV infections after chemoimmunotherapy for EBV-associated nasopharyngeal carcinoma: a case report and literature review
Source: Front Oncol. 2025 Oct 14;15:1608787. doi: 10.3389/fonc.2025.1608787 (PMC12558793; doi:10.3389/fonc.2025.1608787)
Supplement: Supplementary file 1 [file Image1.pdf]

Supplementary Material

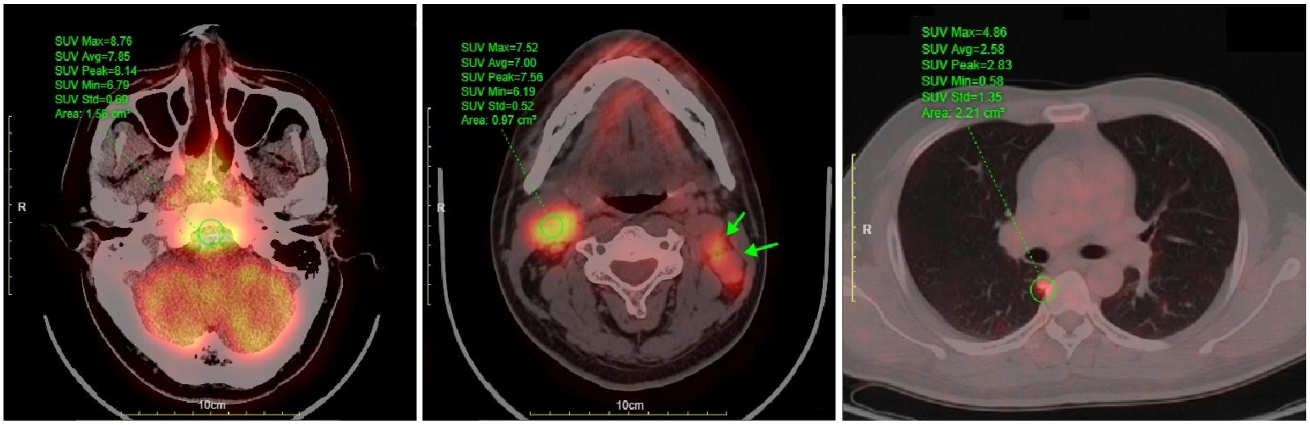

Supplementary Figure 1. PET-CT showed increased uptake in the nasopharyngeal soft tissue mass, bilateral parapharyngeal and cervical lymph nodes, and pulmonary nodule. SUVmax values were 8.76, 7.52, and 4.86, respectively.

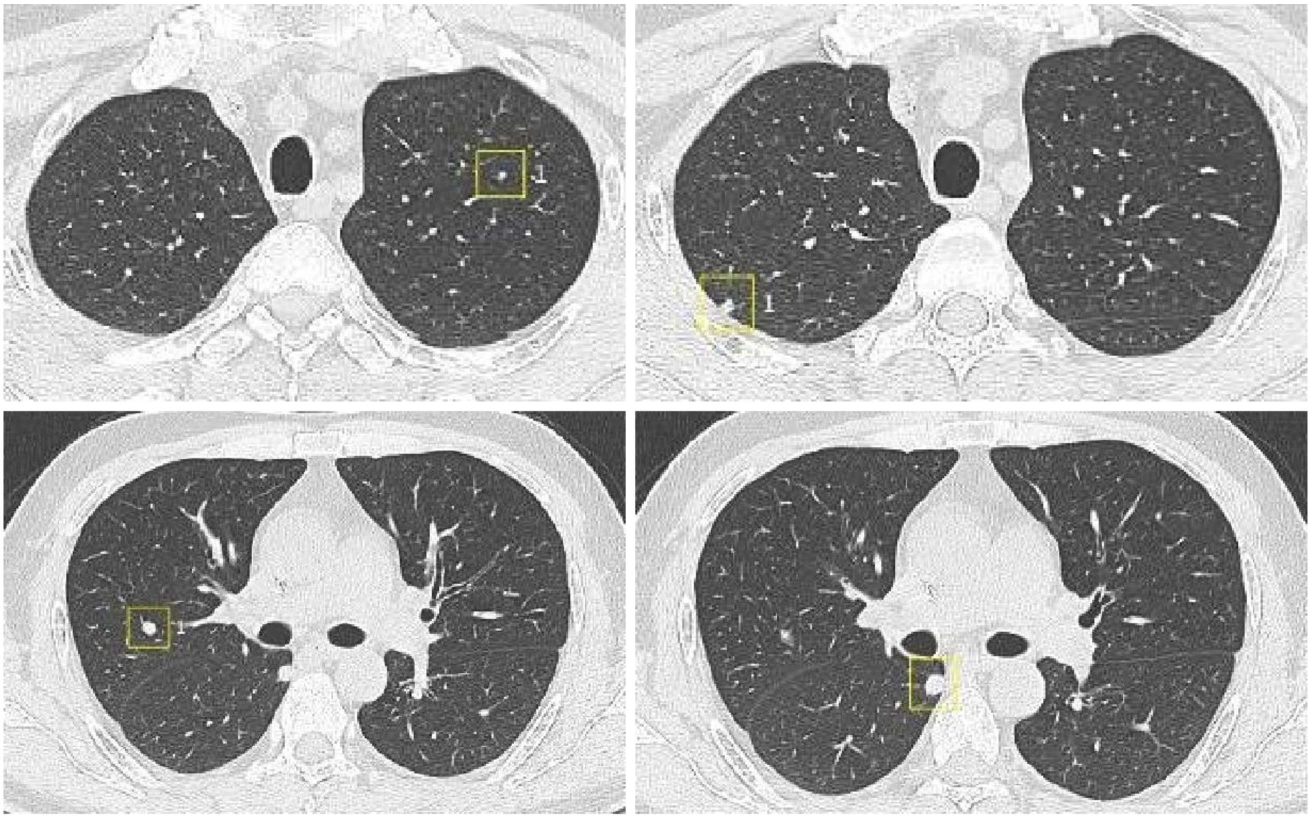

Supplementary Figure 2. Chest CT scan on April 6 revealed multiple solid nodules in both lungs, some of which raised concern for metastatic tumors, along with mild inflammatory changes and

fibrotic lesions in the lingular segment of the left upper lobe and the posterobasal segment of the right lower lobe.

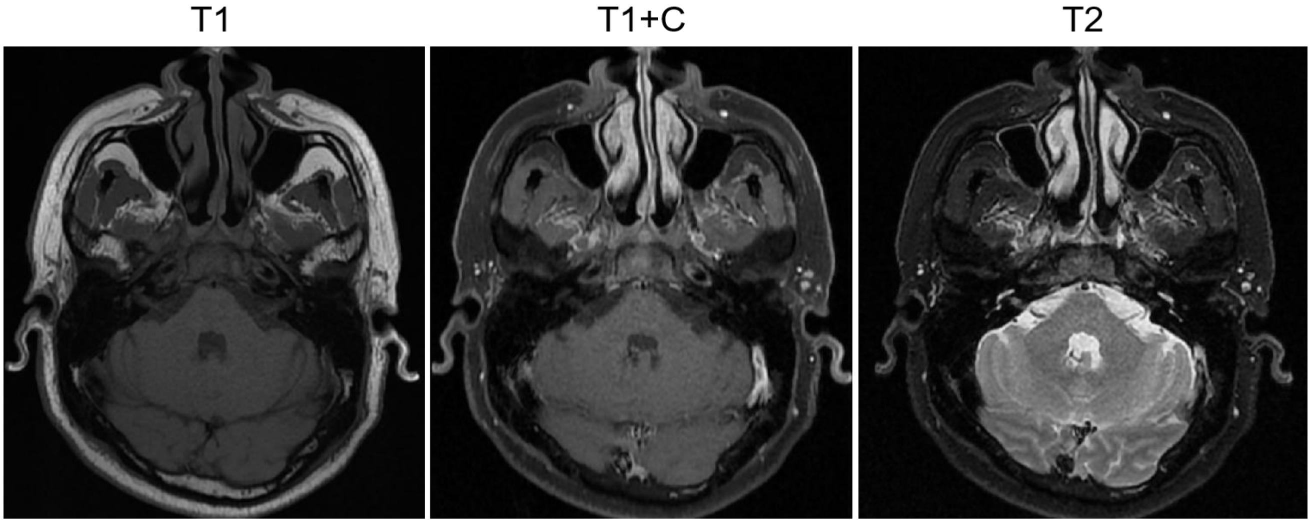

Supplementary Figure 3. The MRI performed on September 27 demonstrated reduced involvement of the surrounding tissues by the nasopharyngeal tumor compared to previous imaging.

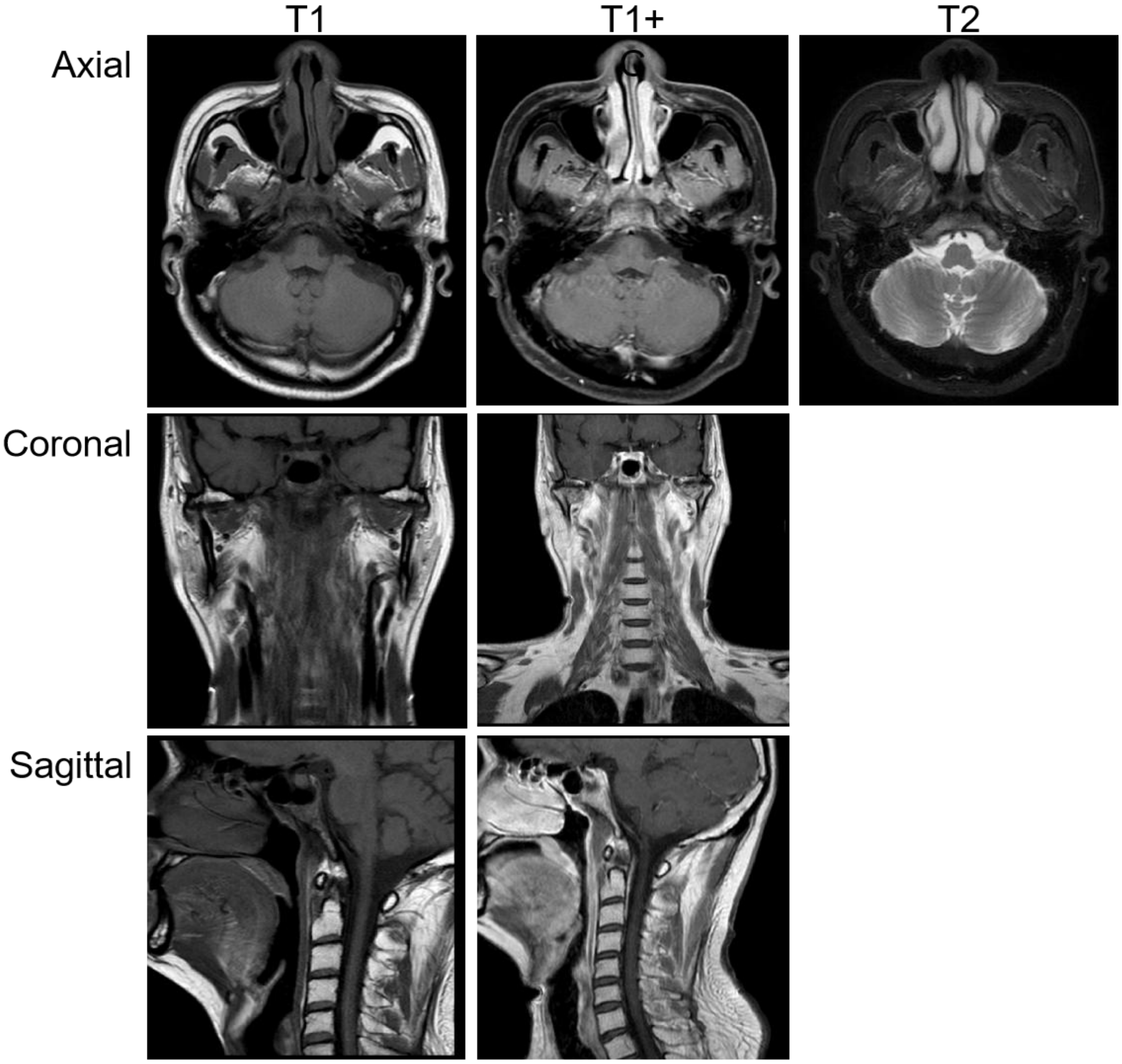

Supplementary Figure 4. The MRI performed on January 16, 2025, demonstrated reduced involvement of the surrounding tissues by the nasopharyngeal tumor compared to previous imaging.
